# Hematopoietic stem cell transplantation (HSCT) as an example of personalized approach in the management of individual disease

**DOI:** 10.1186/1878-5085-5-S1-A62

**Published:** 2014-02-11

**Authors:** Hele Everaus

**Affiliations:** 1 University of Tartu, Tartu University Hospital, Estonia

## 

Hematopoietic stem cells (HSCs) and their transplantation has proved itself as an effective approach to treat different diseases. Since the first 3 cases of successful allo-HCT in 1968, the number of allo-HCTs performed annually has increased steadily over the past 3 decades [1]. From the very beginning HSC demonstrates the importance of individual/personal approach in all steps of the management of the disease. One millionth transplant is celebrated in 2013. Individual treatment plan is unavoidable from the very first days after diagnose. There is necessary to target the cure of the patient. For that purpose right treatment options pretransplant are necessary to choose. Many disease specific transplant regimens are under development to improve transplant outcome after HT. Depending on the disease and patient’s status the source of the HSCs (autologous or allogeneic) will be considered.

## Choice of the stem cell source

Traditionally, hematopoietic stem cells were harvested from the posterior iliac crests under general anaesthesia. More recently, mobilized peripheral blood stem cells have been increasingly used in both auto- and allo-HSCT.

Hematopoietic stem cell transplantation presents a unique opportunity to study the complex and systemic effects of introducing a new genome in an individual. The biological perturbations caused depend on the genetic make-up of the donor and that of the recipient. Validation of the role of each individual HLA locus was established sequentially by comparing populations of patients of different but known genetic composition (monozygotic twins, siblings, haplotype-matched family members and HLA-matched unrelated individuals).

The best donor remains an HLA-identical sibling donor. The best unrelated donor has been shown to be one who is matched, at high resolution for the major polymorphic HLA loci. HLA-A, -B, -C, -DRB1 (8/8) are all considered critical and many would consider an 8/8 matched donor as the gold standard. Genetic disparity in particular at the HLA loci, between patient and donor is a critical factor influencing transplantation outcome.

## Prognostic aspects – predicting the clinical outcome of allogeneic hematopoietic stem cell transplantation

One of the main factors influencing the clinical outcome of allogeneic hematopoietic stem cell transplantation is the restoration of a functional immune system. The post-transplant period is characterized by multiple immune defects that expose the patient to a high risk of opportunistic infections and eventually, disease relapse. The duration of this period may vary according to several variables, including patient’s age and immune status before transplant, the degree of donor compatibility, the intensity of the conditioning regimen, the source of stem cells, graft manipulation, and pharmacological immune suppression. Measurable immune biomarkers predicting the clinical outcome of HSCT await formal validation.

In conclusion, hematopoietic stem cell transplantation has demonstrated well the importance and role of personalized approach in the implementation of this technology in the treatment of different severe diseases. However, in the era of predictive and molecular medicine the practice of HSCT is still characterized by many prognostic uncertainties. Therefore several questions will have to be addressed with the attempt to development approaches including the balance between the effectiveness of the therapy, safety, costs and socio-economic impact.
